# Aerobic Fitness, Micronutrient Status, and Academic Achievement in Indian School-Aged Children

**DOI:** 10.1371/journal.pone.0122487

**Published:** 2015-03-25

**Authors:** Ishaan K. Desai, Anura V. Kurpad, Virginia R. Chomitz, Tinku Thomas

**Affiliations:** 1 Harvard University, Faculty of Arts and Sciences, Cambridge, Massachusetts, United States of America; 2 Division of Nutrition, St. John’s Research Institute, St. John’s National Academy of Health Sciences, Bangalore, India; 3 Department of Public Health and Community Medicine, Tufts University School of Medicine, Boston, Massachusetts, United States of America; 4 Division of Epidemiology and Biostatistics, St. John’s Research Institute, St. John’s National Academy of Health Sciences, Bangalore, India; University of Western Ontario, CANADA

## Abstract

Aerobic fitness has been shown to have several beneficial effects on child health. However, research on its relationship with academic performance has been limited, particularly in developing countries and among undernourished populations. This study examined the association between aerobic fitness and academic achievement in clinically healthy but nutritionally compromised Indian school-aged children and assessed whether micronutrient status affects this association. 273 participants, aged 7 to 10.5 years, were enrolled from three primary schools in Bangalore, India. Data on participants’ aerobic fitness (20-m shuttle test), demographics, anthropometry, diet, physical activity, and micronutrient status were abstracted. School-wide exam scores in mathematics and Kannada language served as indicators of academic performance and were standardized by grade level. The strength of the fitness/achievement association was analyzed using Spearman’s rank correlation, multiple variable logistic regression, and multi-level models. Significant positive correlations between aerobic capacity (VO2 peak) and academic scores in math and Kannada were observed (*P* < 0.05). After standardizing scores across grade levels and adjusting for school, gender, socioeconomic status, and weight status (BMI Z-score), children with greater aerobic capacities (mL * kg-1 * min-1) had greater odds of scoring above average on math and Kannada exams (OR=1.08, 95% CI: 1.02 to 1.15 and OR=1.11, 95% CI: 1.04 to 1.18, respectively). This association remained significant after adjusting for micronutrient deficiencies. These findings provide preliminary evidence of a fitness/achievement association in Indian children. While the mechanisms by which aerobic fitness may be linked to academic achievement require further investigation, the results suggest that educators and policymakers should consider the adequacy of opportunities for physical activity and fitness in schools for both their physical and potential academic benefits.

## Introduction

There is a growing need to improve physical fitness and activity among Indian children [1–3]. The 2013 EduSports Annual School Health and Fitness survey, which studied nearly 78,000 school-aged children in 68 cities and 17 states of India, showed that fitness levels in Indian children are “alarmingly low” in all age groups and regions [4]. While further research on this topic is needed, initial findings suggest that urban Indian children may be less aerobically fit than their rural counterparts [1].

The health benefits of aerobic fitness in children are well documented [5]. Aerobic fitness and activity positively support musculoskeletal, cardiovascular, and mental health and may track into adulthood [6,7]. Developing a fit and active lifestyle during childhood may positively influence health behaviors during adulthood and reduce the risk of noncommunicable diseases [3,8], which accounted for 65 percent of global deaths and 54 percent of global disability-adjusted life years (DALYs) in 2010, according to the Global Burden of Disease Study 2010 [9,10]. Promoting aerobic activity among children is therefore considered an effective strategy for health promotion and disease prevention [7].

Recent findings suggest that the benefits of aerobic fitness may extend beyond overall health and wellbeing. A small but growing body of research has observed that physical fitness is positively associated with academic achievement in school-aged children and youth [11–23]. Studies examining the fitness/achievement association have generally focused on children in developed nations, and no such study has been identified in India, where research on fitness and physical performance among children is relatively limited. In addition, the effect of nutrition and micronutrient status on the association between fitness and academic achievement has not been well studied in any population. Deficiencies in certain micronutrients, such as iron and the B vitamins, may negatively affect cognitive and physical performance and have negative consequences for growth and development in children [24]. This is especially relevant in a developing country like India, where the burden of childhood undernutrition and anemia is high and dietary intakes of several micronutrients are well under their estimated average requirements [24,25].

This study has two main purposes. First, it aims to examine and quantify the relationship between aerobic fitness and academic achievement in clinically healthy but nutritionally compromised school-aged children in urban South India. Second, it seeks to determine how micronutrient status and other potential confounders, including school, age, socioeconomic status (SES), anthropometry, diet, and physical activity, affect this relationship.

## Materials and Methods

This analysis used data from a double-blind, placebo-controlled, randomized trial that examined the impact of multiple micronutrient supplementation on physical performance in school-aged children in Bangalore, Karnataka [26]. Briefly, the intervention was administered to 300 children over a four-month period in 2008 and showed that daily administration of a multi-micronutrient-fortified beverage improved aerobic fitness when compared to a matched, energy-equivalent, unfortified beverage and an untreated control. Data on the participants’ physical performance outcomes, demographics, anthropometry, micronutrient status, diet, and physical activity was collected at baseline and at endpoint. In the current study, endpoint data from the trial was matched with academic performance on school-wide exams and used for cross-sectional analysis to assess potential associations among participants’ aerobic fitness, academic achievement, and micronutrient status.

### Participants

A total of 287 clinically healthy boys and girls between the ages of 7 and 10.5 y completed the previous intervention study and were considered for endpoint assessment. Academic exam results were missing for 14 of these children. Therefore, data from 273 participants was used in the current analysis. Participants eligible for the intervention study had height-for-age and weight-for-age Z-scores of 0 to ≥ -3, were neither severely anemic nor physically disabled, and had no cardiovascular/respiratory disease or recent medical history that would impair their physical performance. Participants were recruited from three primary schools in Bangalore affiliated with the Karnataka State Board and belonged to three socioeconomic groups (upper lower, 215; lower middle, 50; and upper middle, 8), as determined by the modified Kuppuswamy score, which categorizes families’ socioeconomic status based on their education, occupation, and income [27]. In the current study, participants of lower middle and upper middle SES were grouped due to the relatively low number of participants in the upper middle group. Primary data collection and the protocol of the intervention study were approved by the Institutional Ethics Review Board of St. John’s Medical College. Oral consent and written informed consent were obtained from the participants and their parents or legal guardians, respectively

### Aerobic fitness

The primary indicators of aerobic fitness in the intervention study were whole body endurance and aerobic capacity and were therefore used in the current study. Whole body endurance, reported as number of shuttles, was measured by a 20-m shuttle test, in which participants ran back and forth between two markers spaced 20 m apart at a specified minimum pace. The pace was indicated by successive audible beeps. The initial speed was set at 4 km * h^-1^ (1.11 m * s^-1^) and increased by 0.5 km * h^-1^ (0.14 m * s^-1^) every minute. Therefore, the total number of shuttles run by each participant corresponded to the maximum speed he or she attained on the test. Participants ran until exhaustion or until they failed to reach two consecutive markers within the allotted time. The 20-m shuttle test was administered in groups of three to five to improve motivation and was conducted by trained personnel to ensure standardized assessment protocol. Aerobic capacity, defined as the peak oxygen uptake (VO_2_ peak), was estimated using an age-appropriate prediction equation that takes into account the results of the 20-m shuttle test and the ages of the participants [28].

### Anthropometry

Height was recorded to the nearest 0.1 cm using a calibrated stadiometer and weight to the nearest 0.1 kg in school clothing without footwear (Salter Digital Weighing Scale). Body mass index (BMI) was computed as the participants’ weight (kg) divided by the square of their height (m). Z-scores for height-for-age (HAZ), weight-for-age (WAZ), and BMI-for-age (BMIZ) were determined based on WHO growth reference data [29]. Triceps and calf skinfolds were measured and used in a skinfold equation to estimate body fat percentage [30].

### Micronutrient status

Biochemical assessment was conducted at St. John’s Research Institute to determine the post-intervention micronutrient status of the participants. Nonfasting blood samples were collected, and plasma and serum were analyzed for micronutrient markers. Biochemical data was obtained for hemoglobin, RBC riboflavin, plasma pyridoxal phosphate, plasma vitamin B-12, RBC folate, RBC thiamine, niacin, serum vitamin C, plasma ferritin, serum CRP, and plasma soluble transferrin receptor (sTfR). Micronutrient deficiencies in the participants were determined using standard cut-off values. Detailed methodology concerning biochemical assessment and standard cut-off values are found in the original intervention study [26].

### Diet and physical activity

Endpoint data on diet and physical activity was collected through methods similar to those used in previous diet/physical activity studies in urban South India [2,31,32]. Trained interviewers administered a 24-h food recall questionnaire, and the nutrient composition of the diet was estimated using food composition tables to calculate daily energy, protein, fat, and carbohydrate intakes [31,32]. Physical activity was measured through a questionnaire that documented the type and duration of various activities at school and at home, including travel to school, physical education periods, weekend activities, studying, and television viewing [2]. Metabolic equivalent (MET) values were assigned to all documented activities [33,34]. Activities below a MET of 3 were categorized as sedentary activity (SA), while those equal to or above 3 were categorized as moderate to vigorous physical activity (MVPA).

### Academic achievement

Academic achievement was measured through school-wide exams administered by schoolteachers in the three schools from which participants were recruited for the previous intervention study. The exams were administered in compliance with the Karnataka State Board curriculum standards in December 2008, coinciding with the time at which the post-intervention 20-m shuttle test was conducted. Subject areas assessed included mathematics, Kannada (the official language of the Indian state of Karnataka), English, science, and social science. However, the specific combination of subjects assessed varied depending on grade level. Only math and Kannada scores, reported as percentages, were used as final academic outcome measures because these two subjects were assessed in all participants, regardless of grade level. Exam results were de-identified prior to analysis and obtained for all participants who completed the intervention study and completed their math and Kannada exams. The sample of students spanned four consecutive grade levels (Grade 2 to Grade 5).

### Statistical analysis

The primary outcome of interest for this analysis was academic achievement, which was measured by math and Kannada scores. These scores were not standardized across grade levels. Therefore, to allow for inter-grade and inter-age comparison, they were converted into Z-scores using their mean and SD in each grade level. Dichotomous measures of academic achievement were also established, with “academic success” in each subject area defined as a Z-score greater than zero. Dichotomization allowed for more interpretable results since a Z-score greater than zero indicates an above-average academic performance in a given grade level.

Descriptive statistics are reported as mean ± SD for normally distributed data, median (25th percentile, 75th percentile) for non-normally distributed data, and percent frequency (number) for categorical data. The Shapiro-Wilk normality test was used to assess the distribution of continuous variables. Spearman’s rank correlations were computed to determine associations among continuous academic Z-scores, indicators of aerobic fitness, weight status (BMIZ), and micronutrient status. The percentages of students with above-average academic scores—adjusted for gender, school, and SES—were plotted against the highest speed reached on the 20-m shuttle test to visualize trends in the data.

Results from descriptive analysis guided the construction of logistic regression models to evaluate the odds of scoring above average in math and Kannada based on participants’ aerobic fitness. Predicted aerobic capacity (VO_2_ peak) was the primary explanatory variable, and academic success in each subject area (Z-score > 0) was the response variable. Bivariate logistic regression models were initially used to determine the crude odds ratios between aerobic capacity and academic success. Multiple variable logistic models were constructed to examine the association of aerobic fitness with academic success while controlling for confounding variables. Odds ratios with 95% confidence intervals are presented as OR (95% CI). Covariates describing participants’ school, age, demographics, anthropometry, diet, and physical activity were added sequentially to the models and retained if statistically significant (*P* < 0.10). SES and BMIZ were also included in the models regardless of statistical significance since some studies suggest that SES and weight status may be associated with academic performance [12–14,19,20,35–39].

Deficiency status of each micronutrient measured was then added as a binary variable to the multiple variable logistic regression models and retained if statistically significant (*P* < 0.10) to determine whether deficiency in a given micronutrient alters the fitness/achievement relationship. Deficiency variables were included one at a time without other micronutrient covariates so that the effect of each deficiency could be evaluated independently. Only those micronutrient deficiencies that were found to be significant would be included in the final multiple variable logistic regression models. The results of the final adjusted models were confirmed through multi level logistic regression where the clustering of students within 3 schools was accounted for. A random intercept multi level logistic model was used by fitting a two-level model (individuals at level 1 nested within school at level 2). An initial null or unconditional model without any exposure variables was specified to decompose the amount of variance that existed within schools. Thereafter, all variables considered in the multiple logistic regression were included in the multi level model. The improvement in model fit with the addition of variables in comparison to the null model was examined using -2 log likelihood ratio tests with appropriate degrees of freedom. This analysis examined the association between achievement and fitness after controlling for school level clustering.

All analyses were performed using Stata 13 (StataCorp LP, College Station, TX, USA).

## Results

Descriptive endpoint data is presented in Table 1 (n = 273). Half of the participants were female, and approximately 79% belonged to the upper lower socioeconomic group. Participants had average HAZ, WAZ, and BMIZ that were 1.00 SD, 1.36 SD, and 1.16 SD lower, respectively, than the WHO reference values. 9.9%, 19.8%, and 15.4% of participants were considered stunted, underweight, or thin, respectively (results not shown), based on WHO-established cut-off points for children and adolescents of 5 to 19 years [29,40,41]. The median number of shuttles run by participants was 56, and the median predicted aerobic capacity (VO_2_ peak) was 38.9 mL * kg^-1^ * min^-1^. Sample data on the prevalence of micronutrient deficiencies is presented in Table 2. Riboflavin and niacin deficiency were most prevalent, seen in over 50% of participants. Over 10% were deficient in pyridoxal phosphate, vitamin B-12, thiamine, ferritin, and sTfR.

**Table 1:** Characteristics of participants (n = 273 unless stated otherwise).

Characteristics of Participants	Value
Gender (female)	50.2 (137)
Grade Level:	
2	16.1 (44)
3	31.1 (85)
4	24.2 (66)
5	28.6 (78)
School	
Site 1	45.1 (123)
Site 2	13.9 (38)
Site 3	41.0 (112)
Socioeconomic status (upper lower)	78.8 (215)
Age (*y*)	8.83 (8, 9.67)
Height (*cm*)	125 ± 7
Weight (*kg*)	22.3 (19.9, 24.5)
Body mass index (kg * m ^*-2*^)	14.2 (13.5, 15.1)
Body fat (*%*)	12.9 (10.4, 15.8)
Height-for-age Z-score (*SD*)	-1.00 ± 0.75
Weight-for-age Z-score (*SD*)^a^	-1.36 ± 0.83
BMI-for-age Z-score (*SD*)	-1.16 ± 0.95
Energy intake (kJ * d ^*-1*^)	4110 (3300, 5010)
Protein intake (g * d ^*-1*^)	26.1 (21.1, 32.7)
Fat intake (g * d ^*-1*^)	21.4 (14.7, 27.9)
Carbohydrate intake (g * d ^*-1*^)	166 (135, 204)
Moderate to vigorous physical activity (MET * min * wk ^*-1*^)	1320 (817, 2030)
Sedentary activity (MET * min * wk ^*-1*^)	1530 (1180, 2050)
Whole body endurance (*number of shuttles*)	56 (48, 66)
Predicted aerobic capacity, VO_2_ peak (mL * kg ^*-1*^ * min ^*-1*^)	38.9 (35.4, 41.5)

Values are percentages (number) for categorical data, median (Q1, Q3) for non-normally distributed data, and mean ± SD for normally distributed data.

^a^ n = 243

**Table 2:** Prevalence of micronutrient deficiencies (n = 268–273).

Micronutrient, ***Cut-off value***	Percent Deficient
RBC Riboflavin, *Ratio > 1*.*4*	56.6
Plasma Pyridoxal Phosphate, < 20 nmol * L ^*-1*^	10.7
Plasma Vitamin B-12, < 150 pmol * L ^*-1*^	12.1
RBC Folate, < 304 nmol * L ^*-1*^	0.4
RBC Thiamine, < 70 nmol * L ^*-1*^	21.5
Niacin, *number < 100*	69.5
Serum Vitamin C, *< 14*.8 μmol * L ^*-1*^	8.2
Plasma Ferritin^a^, *< 33*.7 pmol * L ^*-1*^	16.8
Plasma sTfR, *> 7*.6 mg * L ^*-1*^	14.3

Values are percentages (% deficient).

^a^ Analysis only for participants with CRP < 47.62 nmol * L^-1^

**Fig 1:**
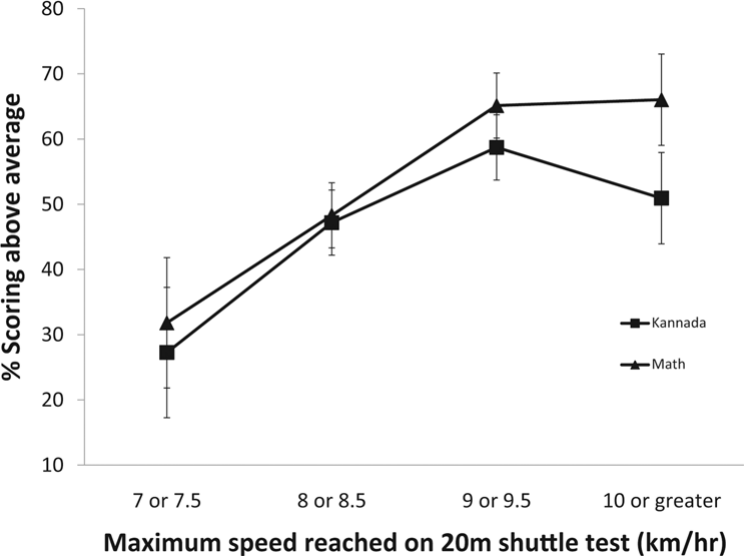
Percentage of participants with above average academic performances by maximum speed reached on 20-m shuttle test. Scoring above average in math (solid triangle) and Kannada (solid square) was defined by an academic Z-score > 0. The initial speed of the 20-m shuttle was set at 4 km * h^-1^ (1.11 m * s^-1^) and increased by 0.5 km * h^-1^ (0.14 m * s^-1^) every minute. Academic Z-scores were adjusted for school, gender, and SES. The percentage of participants with above-average performances in math and Kannada increased as the maximum speed reached increased. The number of participants reaching the above maximum speeds is as follows: 7.0 or 7.5 km * hr^-1^, 22 students; 8.0 or 8.5 km * hr^-1^, 89 students; 9.0 or 9.5 km * hr^-1^, 109 students; 10.0 km * hr^-1^ or greater, 53. Participants reaching maximum speeds of 10.0 km * hr^-1^ or greater were grouped due to the relatively small number of participants who reached speeds above 10.0 km * hr^-1^. Values presented are adjusted mean±SE.

As the maximum speed reached on the 20-m shuttle increased, the number of participants with above-average performances in math and Kannada (Z-score > 0) also tended to increase (Fig 1). Nonparametric correlational analysis revealed significant positive correlations between aerobic fitness indicators and academic Z-scores (Table 3). Academic Z-scores for both math and Kannada were significantly correlated with whole body endurance (ρ = 0.17 and 0.19, respectively, *P* < 0.01) and with aerobic capacity (ρ = 0.14 and 0.14, respectively, *P* < 0.05). BMIZ was not significantly correlated with aerobic fitness indicators or with academic Z-scores.

**Table 3:** Spearman rank correlations between continuous measures of aerobic fitness and academic performance (n = 273).

	Math Z-Score	Kannada Z-Score
Endurance (20-m shuttle)	0.17**	0.19**
Predicted aerobic capacity (VO_2_ peak)	0.14*	0.14*
BMI for age Z score	-0.01	-0.03
Math Z-Score		0.695***

*P < 0.05,

**P < 0.01,

***P < 0.001; two-tailed

Positive associations between aerobic capacity and academic success were confirmed in bivariate logistic regression models of academic success (Z-score > 0). The sequential addition of covariates for each micronutrient deficiency did not alter these associations (OR did not change), nor did the cumulative number of deficiencies out of the nine considered in this study. Covariates for diet (daily energy intake, protein intake, fat intake, carbohydrate intake) and physical activity (SA, MVPA) were also not significant predictors in the regressions. The final multiple variable logistic regression models (Table 4), adjusted for participants’ school, gender, SES, and BMIZ, estimated that a one-unit increase in aerobic capacity (mL * kg^-1^ * min^-1^) increased the odds of scoring above average in math and Kannada by 8% and 11%, respectively. Odds of success in Kannada were significantly higher in females than in males. SES and BMIZ were not significantly associated with academic success in either subject area. The percentage of variability accounted for by clustering in school, represented as the variation proportion coefficient in the null model was 12% for Kannada and 13% for Math. With the inclusion of the individual level variables in the model, this variability did not change and the OR obtained confirmed the results of logistic regression while accounting for within school clustering of participants (results not presented).

**Table 4:** Final logistic regression models predicting odds of academic success in math and Kannada from aerobic fitness (n = 273).

	Odds of Success in Math	Odds of Success in Kannada
Aerobic capacity (*mL* * *kg* ^*-1*^ * *min* ^*-1*^)	1.08 (1.02–1.15)*	1.11 (1.04–1.18)**
School	0.57 (0.44–0.75)***	0.56 (0.42–0.74)***
Gender (female)	1.04 (0.62–1.77)	1.90 (1.10–3.27)*
Socioeconomic status (upper lower)	1.18 (0.64–2.18)	0.97 (0.52–1.80)
BMI-for-age Z-score (SD)	0.91 (0.69–1.20)	1.05 (0.79–1.39)

Odds ratios are presented as point estimates (95% confidence interval) and are adjusted for school, gender, SES, and BMIZ. Academic success in math and Kannada is defined as a Z-score > 0.

*P < 0.05;

**P < 0.01;

***P < 0.001

## Discussion

The main finding of this study is that aerobic fitness is positively associated with academic achievement in school-aged children in urban South India. Associations with both whole body endurance and aerobic capacity were noted using either continuous or dichotomous academic scores. Significant odds ratios confirmed that aerobically fitter children were more likely to score above average on school-wide exams in math and Kannada, even after controlling for factors such as gender, school, SES, anthropometry, diet, physical activity, and micronutrient status. The findings contribute to a small but growing collection of studies that have observed modest positive associations between physical and academic performance in children and adolescents [11–23]. Studies have varied by design, methodology, choice of fitness and academic variables, control variables, and overall conclusions. Nevertheless, most have found one or more positive relationships between the fitness and academic variables they selected. Some studies used FITNESSGRAM (Cooper Institute, Dallas, Texas, USA) as their measure of physical fitness. FITNESSGRAM is a test battery that evaluates multiple components of health-related fitness, including aerobic fitness, body composition, muscular strength, muscular endurance, and flexibility [42]. Of the studies that analyzed individual FITNESSGRAM components [11,12,14,21–23], nearly all found aerobic fitness to be the component most strongly associated with achievement. These findings justify the selection of cardiorespiratory fitness, as measured by the progressive shuttle run, as our primary measure of physical performance.

This study has several strengths. To the best of our knowledge, it is the first to evaluate the fitness/achievement relationship in Indian children and to examine the association of micronutrient status. Second, it included potential confounders that many studies have not considered, including school-level random effects, gender, SES, anthropometry, physical activity, and macronutrient intakes. Third, the study analyzed whole body endurance and aerobic capacity (VO_2_ peak) as continuous explanatory variables rather than as dichotomous variables. In studies that use the FITNESSGRAM test battery, fitness scores that meet criterion-referenced health standards are categorized as “Healthy Fitness Zone”, while those falling below the standards are categorized as “Needs Improvement” [42]. Using such dichotomous indicators of fitness may exclude important variability and observations in the data.

This study also has limitations. First, the cross-sectional design of the study limits our ability to make assumptions about the causal nature of the fitness/achievement relationship. Nevertheless, the logistic models used to assess the odds of obtaining above-average academic performances include a number of potential confounders that are consistent with previous literature on the topic, including SES and weight status (BMIZ). Second, because data was drawn from a previous intervention study, the final sample size (n = 273) was relatively small compared with those of other cross-sectional studies on this topic. Third, although physical fitness was analyzed using a 20-m shuttle test and quantified as continuous variables, physical activity was measured through a questionnaire and metabolic equivalent (MET) values were assigned to each activity. Thus, it is likely that physical activity measurements were subject to reporting biases and misclassification. Finally, the sample was made up of children from only three board-affiliated primary schools, and it was relatively homogeneous with regards to SES, anthropometry, and nutritional status (recruiting those with HAZ and WAZ of 0 to ≥ -3). Therefore, the results may not be generalizable to all Indian school-aged children.

Unique to this study was the investigation of whether micronutrient status influences the association between aerobic fitness and academic achievement. Several participants in the study were nutritionally compromised, as shown by the presence of multiple micronutrient deficiencies (Table 2). A recent review on micronutrient deficiency in Indian children noted that deficiencies in iron and B vitamins may negatively impact functional performance [24]. There were modest correlations of vitamin B12 and vitamin B2 status with aerobic fitness and of vitamin B6 status with academic performance. However, none of the micronutrient deficiencies analyzed in the current study, including plasma ferritin and B vitamins, were found to significantly confound the fitness/achievement relationship in the final logistic models, and the number of deficiencies in a child was not associated with aerobic fitness or academic performance. This suggests that the link between aerobic fitness and academic achievement may exist due to mechanisms that are unrelated to a child’s micronutrient status. Given that the original intervention study enrolled nutritionally compromised children with HAZ and WAZ of 0 to ≥ -3, it is possible that the sample lacked sufficient variability in nutritional status to demonstrate possible associations between micronutrient status, fitness, and achievement.

SES and weight status (BMIZ) had no effect on the fitness/achievement relationship and did not significantly predict scoring above average in either math or Kannada. Studies that account for these variables have yielded mixed results. Some suggest that children of lower SES are more likely to perform worse in certain subject areas [13,14,19,35,39]. Coe et al. [14] found that although both fitness and SES are significantly associated with academic achievement, SES is more strongly associated. There are several possible reasons why this association was not observed in the current study. The Kuppuswamy’s Socioeconomic Status Scale, which was used to determine SES, is specific to India [27]. Most studies on this topic generally examined populations in the United States and used SES proxies such as student eligibility for free or reduced-price lunch in schools. Comparisons should thus be made with caution. Additionally, the sample did not exhibit much socioeconomic diversity. 215 participants belonged to the upper lower socioeconomic group, accounting for nearly 79% of the total sample. It is possible that different results could have been obtained for SES if the sample were larger and more representative of other SES levels.

A negative association between weight status and academic achievement, documented by a few studies [12,20,35,37], was also not apparent. It is important to note, however, that such studies were conducted in the context of childhood overweight and obesity and examined participants representing a broad range of weight statuses. In contrast, the current study specifically examined participants with low HAZ and WAZ according to international reference values. This resulted in a low mean BMIZ (-1.16 ± 0.95), and no participants were considered overweight or obese. Therefore, the potential influence of weight status on academic performance may not be applicable to the sample considered in this study.

The specific mechanisms by which aerobic fitness is linked to academic achievement have not yet been determined. However, there are many plausible explanations. Aerobic fitness has been linked to cognitive and executive function in children and young adults [43–45]. Hillman et al. [45] showed that preadolescent children who were more aerobically fit exhibited higher neuroelectric activity related to attention and working memory, response speed, and cognitive processing speed. Although data among Indian populations is limited, preliminary research has documented similar relationships in Indian children; a study that examined school-aged children in urban South India observed a significant association between aerobic fitness and performance on cognitive tests for comprehension and block design [46]. Physical activity and fitness can also have beneficial effects on concentration, mental health, stress, anxiety, depression, and self-esteem, all of which may contribute to academic success [11,13,22,47–50]. It is unclear which mechanisms have the greatest implications for academic performance, and more research is needed to determine the physiological and psychological basis of the fitness/achievement relationship. Additionally, further research with larger samples is needed to confirm the fitness/achievement relationship in Indian children and youth. Longitudinal and interventional studies should be designed to shed light on how changes in children’s aerobic fitness relate to or directly affect their academic performance.

Schools can have effective settings for improving physical activity levels among children [51]. However, implementation of school-based physical education according to official policies and expectations is low worldwide [52]. In India, national recommendations on health and physical education programs are not consistently followed [53], and many schools and after-school activities deprioritize such programs in favor of other educational objectives. Our findings provide preliminary evidence against this approach. The positive relationship between aerobic fitness and academic achievement in Indian children raises the possibility that aerobic exercise programming could yield benefits for not only child health, but also performance in the classroom.
